# Drug Target Optimization in Chronic Myeloid Leukemia Using Innovative Computational Platform

**DOI:** 10.1038/srep08190

**Published:** 2015-02-03

**Authors:** Ryan Chuang, Benjamin A. Hall, David Benque, Byron Cook, Samin Ishtiaq, Nir Piterman, Alex Taylor, Moshe Vardi, Steffen Koschmieder, Berthold Gottgens, Jasmin Fisher

**Affiliations:** 1 Department of Applied Mathematics and Theoretical Physics, University of Cambridge, Cambridge CB3 0WA, UK; 2 Microsoft Research, Cambridge CB1 2FB, UK; 3 MRC Cancer Unit, University of Cambridge, Cambridge, CB2 0XZ, UK; 4 Department of Computer Science, University College London, London, WC1E 6BT, UK; 5 Department of Computer Science, University of Leicester, Leicester, LE1 7RH, UK; 6 Department of Computer Science, Rice University, Huston 77005-1892, Texas; 7 Department of Medicine, University Hospital of Aachen, Aachen D-52074, Germany; 8 Cambridge Institute for Medical Research, University of Cambridge, Cambridge CB2 0XY, UK; 9 Wellcome Trust and MRC Cambridge Stem Cell Institute, Cambridge CB2 0XY, UK; 10 Department of Biochemistry, University of Cambridge, Cambridge CB2 1GA, UK

## Abstract

Chronic Myeloid Leukemia (CML) represents a paradigm for the wider cancer field. Despite the fact that tyrosine kinase inhibitors have established targeted molecular therapy in CML, patients often face the risk of developing drug resistance, caused by mutations and/or activation of alternative cellular pathways. To optimize drug development, one needs to systematically test all possible combinations of drug targets within the genetic network that regulates the disease. The BioModelAnalyzer (BMA) is a user-friendly computational tool that allows us to do exactly that. We used BMA to build a CML network-model composed of 54 nodes linked by 104 interactions that encapsulates experimental data collected from 160 publications. While previous studies were limited by their focus on a single pathway or cellular process, our executable model allowed us to probe dynamic interactions between multiple pathways and cellular outcomes, suggest new combinatorial therapeutic targets, and highlight previously unexplored sensitivities to Interleukin-3.

Cancer is recognized as a highly complex aberrant cellular state where initiating mutations impact either directly or indirectly on a multitude of regulatory pathways. Chronic Myeloid Leukaemia (CML) represents a paradigm for cancer, both in terms of understanding the nature of the molecular lesion as well as the ability to develop targeted therapies. Whilst the development of targeted drugs has revolutionized the treatment of CML patients, drug resistance is an inevitable consequence of this therapeutic approach. Hence, devising strategies to delay or overcome drug resistance becomes a major challenge, calling for systematic screening of multiple drug targets and their combinations.

Traditionally, biological and medical research has focused on the study of individual genes and proteins in isolation from other elements that comprise the entire system in which they interact and function. While this reductionist approach has been effective in elucidating specific characteristics of particular biological processes, scientific discovery is increasingly limited rather than guided by reductionist principles because the functionality of biomolecules critically depends on interactions with many other biomolecules^1^. Importantly, innovations in high-throughput data generation and automation have set the scene for more integrative approaches^2^. No less important than the generation of data describing biological functional relationships is our ability to interpret this data. Mechanistic diagrams have been commonplace in biology, but these static representations fail to capture variations in relationships over time and the sheer scale of the systems represented often proves these to be too unwieldy. Modeling, and especially computational modeling, has thus become a powerful tool in this endeavor.

While mathematical models can be simulated through translating mathematics to algorithms, computational models are immediately executable, allowing for larger-scale simulation of biological systems^3^. In addition, analysis techniques common in computer science and formal verification can be directly applied to such models. One such technique, model checking, involves analyzing all possible executions of the model, but without actually executing all these possibilities^4^. This analysis allows for rapid and thorough comparison of the computational model with experimental data; a cyclic process is thus able to be realized in which a draft model is composed, model checking is applied, the model is assessed to see if it fits with experimental data, and a revised model is produced. Boolean networks, pioneered by Kauffman as a model for genetic regulatory networks, have already been used in interpretation of large data sets as well as for drug discovery^5^,^6^,^7^. In this formalism, relationships are represented in a dynamic network with discrete time steps. Genes in this type of networks, represented by nodes, can have two states (hence a Boolean network) and edges are directed and may be activating or inhibitory.

In this study, we use the *Qualitative Networks* (QNs) generalization of Boolean Networks^8^ to model the gene regulatory network of CML. CML has been extensively mathematically modeled on a cell population level, but not at the level of a genetic network^9^,^10^,^11^. CML represents an ideal model for the genetic study of malignancy, since it is linked to a consistent molecular event, the translocation between chromosomes 9 and 22, which gives rise to the so-called Philadelphia chromosome expressing the oncogenic fusion protein Bcr-Abl. If untreated, CML has a well-defined and mostly-uniform progression from the relatively manageable chronic phase (CP) to its terminal blast crisis (BC) phase^12^. In this work we first integrated the current body of knowledge on the molecular pathways involved in CML into a gene regulatory network via manual inspection of the relevant literature. We then constructed a Qualitative Network executable model of CML progression using the BMA tool (freely available at http://biomodelanalyzer.research.microsoft.com/) based on the CML network curated from the literature. The analysis of our CML network-model had generated novel hypotheses for network sensitivity via *in silico* removals and knock-outs of combinations of cytokines, genes, and genetic interactions (Figure 1). Furthermore, the model suggested new combinatorial therapeutic targets and highlighted unknown sensitivities to IL-3. Further enhanced by the user-friendly interface of BMA, this study serves as a proof of concept for the wider community on how the implementation of state of the art computational modeling can become a routine procedure for the whole of the modern biomedical research community.

## Methods

### Qualitative Networks

Qualitative networks (QN) extend Boolean networks by allowing variables to range over larger discrete domains and replacing Boolean functions by algebraic functions^8^. Intuitively, a Qualitative Network associates a discrete variable with every substance the model follows. The variable ranges over a small discrete domain where values represent expression levels of the substance such as {0 = off, 1 = low, 2 = medium, 3 = high}. For every substance, a target function reads the values of other substances that affect it. The target function sets a value to which the substance should get to eventually. The substance changes gradually to attain this target.

More formally, a Qualitative Network is defined as follows. A Qualitative Network is *Q* = (*V*, *T*, *N*) consists of a set of variables *V* = {*v*_1_,…, *v_n_*} ranging over {0, …, *N*} and a set of target functions T = {*T*_1_, …,*T_n_*}. A state of the network is *s*: *V* → {0, …, *N*}, i.e., a valuation for all the variables in *Q*. A target function *T_i_* associates with a state *s* a value in {0, …, *N*}, the value towards which *v_i_* should move from state *s*.

A Qualitative Network gives rise to a transition system between its states. As explained, the values of substances/variables change by pursuing their targets gradually, i.e., they change by at most 1 in every transition. Thus, every state *s* = {*d*_1_,…, *d_n_*}, where the value of *v_i_* is *d_i_*, has a successor *s*′ = *T*(*s*), where *s*′(*v_i_*) is defined as follows:
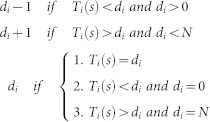


Notice that all variables are updated simultaneously. A discrete change in the value of a variable corresponds to a number of simultaneous molecular events over a period of time, and as such non-deterministic events are not considered. Target functions default to the difference between the average of the activator nodes and the average of the inhibitor nodes unless explicitly altered.

It follows that a Qualitative Network defines a finite state transition system where state changes are deterministic. Then every execution of a Qualitative Network ends in a cycle of states that are visited infinitely often. We say that a state is *recurring* if it appears in an execution that starts from itself. That is, if for some finite number of applications of *T*, we have *s* = *T_i_*(*s*). A network is *stabilizing* if there is a unique state *s* such that *s* = *T*(*s*) and no other state is recurring.

### The BioModelAnalyzer (BMA) Platform

BMA is a graphical tool for the construction and analysis of biological models in the form of *Qualitative Networks* (QNs). It uses visual notations to implicitly define QNs, and has been designed with and for users who have no experience with computational modeling and analysis. Models are built up on a gridded canvas, onto which cells, cell elements, and connections between them can be dragged and dropped. Modeling starts by adding cells onto the grid. Though cells do not have a functional role in the formal mathematical definition of the model they do make the model more visually appealing and make it easier to design models that have repeating motifs in them (by copying and pasting whole cells). Then proteins or genes are added to the cells, each corresponding to a variable in the underlying QN. There are two types of proteins: membrane bound receptors and standalone proteins, which lie inside the cell. Proteins can be placed inside and outside cells, and can be set to constant values. Activation (arrows) and inhibition (bar arrows) influences can be drawn between proteins or genes (Supplemental Figure 2a).

The drawing created using the graphical user interface gives rise to an underlying QN. Initially, all values of this QN are default with the default range of {0, 1} per every protein, and the default target function of “average difference” (i.e., representing the average of activation influences minus the average of inhibition influences), as illustrated in Supplemental Figure 2B. Next, the QN can be further specialized using simple drop-down menus where protein names, ranges, and target functions can be defined. Target functions can be composed using standard algebraic and numerical operations (such as addition, subtraction, mulitplication, division, minimum, maximum, average) and by referring to constants or other proteins that are affecting this protein (i.e., having an arrow or bar-arrow to it) using the keyword var(protein_name), thus enabling the straightforward encoding of complex dependencies between the proteins.

BMA currently supports both simulation and stabilization analysis. In a simulation the tool shows an execution of the network starting from a given valuation for the proteins. It is possible to randomize the initial state or choose it (or some of it) directly. A simulation can be complemented by a graph highlighting some of the proteins and drawing their changes over time. Stabilization analysis in BMA is based on the algorithm in Ref. 13. The tool tries to prove that the network does stabilize. If it succeeds, it shows the values on which each of the proteins stabilizes. If the tool cannot establish stabilization, it tries to find executions that will show that the network does not stabilize: either two different executions leading to two different loops or an execution ending in a loop that is larger than 1. The result of stabilization analysis is displayed on the main graph of the network. In case of instability an execution of the model exhibiting the instability is shown.

## Results

### Construction of a quantitative network model for CML

**Figure 2:**
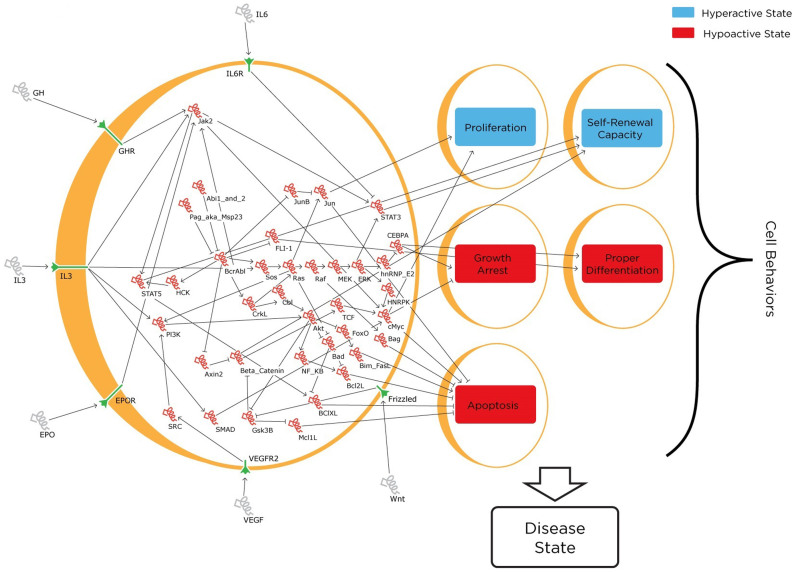
Model of genes, external factors, receptors, and cell behaviors involved in CML. Network visualization through the GUI interface of BMA. External cell factors, receptors, and internal genes are depicted in grey, green, and red respectively.

Based on a manual curation of 160 publications, we constructed a QN model representing a generally accepted canonical set of 37 genes, 6 receptors, and 2 cytokines involved in CML containing a total of 54 nodes (including 4 ligands and 5 cell behaviors) linked by 81 interactions (Supplemental Tables 1, 2, and 3). Each terminally downstream node, defined as those nodes without other nodes further downstream of them, was then categorized into either activating or inhibiting one of five cell fates or behaviors: proliferation, self-renewal capacity, growth arrest, proper/correct differentiation, and apoptosis. Intuitively, the level of proliferation, self-renewal, growth arrest, differentiation and apoptosis corresponds to the existence of markers in the cell that are linked to these behaviors. Iterative modification of the target functions of each node was then performed to ensure that values of individual genes and cell behaviors were in correspondence with actual experimental data (Figure 2).

All nodes were assigned a range of 0 to 2. The decision to restrict the range to these values was made based on the literature survey. Based on the manual curation of the literature, the values represent the following. For genes, 0 represents low or no activity, 1 represents moderate activity, and 2 represents high activity. For the cell fates, 0, 1, and 2 represent absence, moderate amounts of, and high levels of proliferation, self-renewal capacity, and apoptosis respectively. As growth arrest and correct differentiation are conditions that can be represented by a binary variable, a value of 0 corresponds to absence of the cell property, while a value of 1 corresponds to presence of the property. For consistency, the ranges of these two cell fates are kept at 0 to 2, but a value of 2 is not reached in any of the *in silico* experiments or runs of the model. The default target function of “average difference” was assigned to most genes with the modification of rounding the result to the nearest integer instead of rounding to the integer below. For genes with only negative influence, we set their target function to a maximum attainable level (could be 1 or 2) minus the average of the incoming negative influences. This corresponds to being constitutively active unless some inhibition is present. As an additional example, the target function of β-catenin is set to the level of Bcr-Abl + 1 minus the sum of Axin2 and Gsk3B, that is var(Bcr-Abl) + 1-var(Axin2)-var(Gsk3B). From biological literature it is known that that Axin2 is negative regulator of canonical Wnt/TCF signalling by enhancing formation of the β-catenin destruction complex^14^. Also GSK3B phosphorylates β-catenin on the N-terminal Thr41, Ser37, and Ser33 residues, and phosphorylated β-catenin is ubiquitinated by the F-box-containing protein β-TrCP ubiquitin E3 ligase to be degraded by the proteasome.

The non-disease control state was modeled by setting the target function of Bcr-Abl in the model to 0. The CP disease state was modeled by setting the initial value of Bcr-Abl to 2, and setting the initial value of β-catenin to 0, as this pathway is described in the literature as not being activated until BC. The BC disease state was modeled with the initial value of Bcr-Abl at 2, and β-catenin unrestricted.

The model was initially checked without any knock-outs or modifications, and with all factors at the moderate level, 1. The baseline resultant cell behavior levels were as follows: for WT, growth arrest at 0, self-renewal capacity at 1, apoptosis at 1, proliferation at 1, and correct differentiation at 1; for CP CML, growth arrest at 0, self-renewal capacity at 1, apoptosis at 0, proliferation at 2, and correct differentiation at 0; for BC CML, growth arrest at 0, self-renewal capacity at 2, apoptosis at 0, proliferation at 2, and correct differentiation at 0. The resultant cell behaviors therefore correlated well with the known biology of CML with a gradual increase of self-renewal capacity and proliferation accompanied by a simultaneous decrease in apoptosis during the progression from wild type, to chronic phase to blast crisis^12^.

### A 54-node executable model of CML replicates experimental results found in the literature

We next evaluated the accuracy of the 54 node/81 edge network model of CML described above by replicating *in silico* a sample of previous experiments found in literature that were not used to construct or refine the model (Table 1). In six out of seven cases, the model accurately and directly recapitulated the experimental results found in literature. For instance, Sonoyama et al. observed that expression of a single dominant negative form of RAS, STAT5 or PI3K did not induce apoptosis in K562 cells, but expression of combinations of these proteins resulted in caspase-3 activation as measured by FACS^15^. In the *in silico* experiment, the same result is observed: setting the target function of one of RAS, STAT5, or PI3K does not result in an increase of the “apoptosis” cell fate from its base level of 0, but setting the target function of any two of the genes results in an increase from 0 to 1. Holtz et al. observed that treatment of both CML and normal primitive progenitors with Imatinib resulted in a significant decrease of dividing progenitors^16^. In the *in silico* experiment, treatment with Imatinib, simulated by reducing the base constant of Bcr-Abl from 2 to 1 results in a decrease of the “proliferation” cell fate from 2 to 1. The other four successfully replicated experiments included the observations that MEK-ERK activation of HNRPK is not required for cytokine independent proliferation, that Interleukin-3 (IL-3) upregulation induces factor independent growth in leukemic cells, that Bcl-XL suppression induces apoptosis in CML cells, and that Bcr-Abl conveys the ability for CML cells to have factor-independent growth^17^,^18^,^19^,^20^. Experimental results were not exactly duplicated in one case concerning a study by Uchida et al., who had observed that EPO-mediated reduction of apoptosis is increased by Imatinib as measured by Annexin V staining and FACS^21^. In the *in silico* experiment, decreasing EPO from its base level of 2 to 1 resulted in the model remaining at the baseline. A manual inspection of the model under each of these conditions showed that the levels of individual proteins associated with apoptosis were increased. The individual changes however were insufficient to alter the global “apoptosis” aggregator behavior. We therefore conclude that the model accurately reproduces the behavior of individual apoptotic pathways but that the change is small relative to the overall granularity of the cell behavior. This apparent difference may reflect either the model itself, or the specific sensitivities of the different experiments used to determine the activity of apoptotic pathways.

### Simulation of combinatorial gene knock-outs identifies concurrent targeting of Ras and Bcl-xL as a potential strategy to restore normal behaviour in CP CML while limiting negative effects on normal cells

Having demonstrated faithful reproduction of known experimental phenotypes, we next set out to analyze phenotypes following systematic network perturbations. A knock-out of a node in the QN represents the reduction of activity of a protein through non-specific inhibition of the protein activity. For example, Imatinib action represents one possible type of node knock out, as it reduces the kinase activity of Bcr-Abl for all its phosphorylatable substrates. Combinatorial therapy has been identified as a powerful technique for treating diseases whilst minimizing the opportunities for drug resistance and side effects^22^,^23^. It is used therapeutically in diverse diseases, including hypertension^24^, breast cancers^25^ and diabetes^26^. Furthermore, theoretical approaches have been shown previously to be a valuable approach, both for identifying useful combinations and testing a models validity^27^,^28^. We initially focused on all possible pairwise knock-outs of genes. The results are summarized in Table 2 and Supplemental Table 4. Knock-outs of genes were simulated by restricting the range of the gene to a single value 0. Notably, the single knock-out pair, *Ras* and *Bcl-xL*, resulted in the restoration of CP-CML cells to a near-normal state (apoptosis back to normal level of 1) without negatively affecting the WT behavior (all cell fate/behaviors remained at the non-KO baseline levels). In both the CP-CML state and the BC-CML state, the most prevalent effect of a pairwise KO is to remain at their baseline levels, thus indicating that even during the chronic phase, CML represents a robust cellular state, where multiple redundancies in growth and survival pathways allow for the spread and survival of diseased cells.

### CP CML and BC CML resist pairwise removal of gene interactions

Similar to the network perturbations with the pairwise knock-outs of entire gene nodes, we next analyzed pairwise KOs of interaction edges. In contrast to knock-outs of whole nodes, these represent the disruption of specific interfaces between different genes or proteins. A real-world therapeutic example (taken from a different system) of such an effect would be the disruption of the p53-MDM2 interface by Nutlin compounds. Knock-outs of interactions were simulated by setting the value of the specific edge to 0. As summarized in Supplemental Table 5, out of a total of 6240 perturbations that do not alter an edge directly connected to a fate, only five pairs of interaction KOs showed an increase of growth arrest alongside a reduction of proliferation in either the CP CML or the BC CML states. For the KOs of IL3 to IL3R and Bcr-Abl to CrkL, proliferation was reduced in the CP CML state and apoptosis induced, consistent with results found by Seo et al^29^. Proliferation was slowed in the BC CML state from 2 to 1 and apoptosis increased to 1, but otherwise remained the same as the baseline BC CML. For the KOs of Bcr-Abl to Abi1 and Abi2 and Bcr-Abl to CrkL, WT cells remained at their baseline cell behaviors, while CP CML cells reduced both proliferation and self-renewal capacity levels to 1 (but apoptosis remained at 0) and BC CML cells reduced the proliferation (and had apoptosis at 0). Collectively, the results of Supplemental Table 5 are again consistent with significant stability of the CP-CML and BC-CML regulatory states as all are found to be stable. For the KOs of Bcr-Abl to CrkL and Bcr-Abl to Pag, WT cells remained at the normal baseline behavior, while CP CML cells reduced both proliferation and self-renewal capacity levels to 1 (with apoptosis remaining at 0) and BC CML cells reduced proliferation level to 1 (with apoptosis remaining at 0).

### CP CML and BC CML may lose sensitivity to interleukin-6, erythropoietin and growth hormone in comparison to the WT population

In a further set of *in silico* experiments, we next explored the consequences of changing the levels of external factors. Factor levels were varied between 0 and 2 in all possible combinations in the models describing WT cells, CP CML, and BC CML. Growth factor removal was simulated by restricting the range of the factor from 0 to 0, essentially setting the target function to a constant 0. In WT cells, it was found that removal of IL-6 resulted in low self-renewal capacity (0). Pairwise removals of IL-3 with either EPO or GH resulted in growth arrest (1), and low self-renewal capacity and proliferation (0). Removal of EPO and GH together lead to a reduction in self renewal (0). In contrast, in both the CP CML and BC CML states, interestingly only IL-3 removal (with or without removal of EPO and GH) had any effect on cell behavior, resulting in a reduction in proliferation from 2 to 1. This is consistent with previous work by Otsuka et al. indicating that IL-3 can enhance the proliferation of early human hematopoietic cells^30^. These results seem to be in agreement with the consensus in literature that as the disease progresses, cells lose their reliance upon external factors for growth and survival, and instead are driven by Bcr-Abl and the aberrantly-stimulated downstream pathways.

### Imatinib inhibition of Bcr-Abl partially restores the healthy state.

We simulated Imatinib inhibition of Bcr-Abl by reducing the constant in the target function of Bcr-Abl from 2 to 1 (as illustrated in Figure S2). This leads to the model becoming unstable. In this case, instability is associated with the cellular model remaining in a diseased state, but the opposite, where stability is associated with a disease state and instability a shift toward a normal phenotype, is seen in the results described by Holtz et. al. in 2002 where treatment with Imatinib reduces proliferation of progenitor cells^16^. In addition to the expected drop in the Proliferation phenotype node from 2 to 1, the Apoptosis and Correct Differentiation nodes shift from the stable values of 0 to oscillating between values 0 and 1, illustrating that stabilization does not necessarily correspond with the “normal” or “correct” phenotype.

### Combined treatment with Imatinib and a Bcl Family Member Inhibitor mostly restores normal behaviour in the CP CML state without negatively affecting the behaviour of WT cells

**Figure 3:**
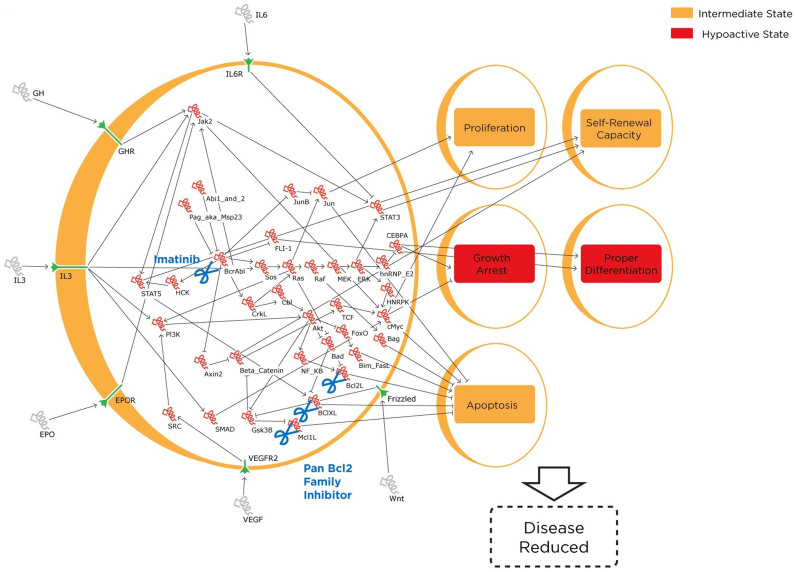
Drug simulation through fixing representative node values. Drug simulation through fixing representative node values. In this model, drugging of the base CP CML model previously illustrated in Figure 2 is simulated by reducing the constant value portion of Bcr-Abl from 2 to 1 (the in silico Imatinib treatment), and preventing Bcl2, Mcl1L, and BclXL from fluctuating to the high level of 2 by fixing these nodes at a constant moderate level of 1 (the in silico pan Bcl2 family member inhibitor treatment). Corresponding changes in phenotype value are illustrated on the right.

Finally, as a brief survey of possible chemotherapeutic options of Imatinib and inhibitors currently available, combinations of “Imatinib treatment” (setting the base Bcr-Abl level to 1) with inhibition of several combinations of genes known to have functioning inhibitors were performed. A total of 175 simulations were performed. Of these, only the Imatinib and pan-Bcl2 family gene inhibitor combination described by Meng *et al.* and Goff *et al.* restored the normal phenotype in the CP CML state without changing the WT population from the baseline state^31^,^32^. Bcl2 family gene inhibition was simulated through fixing Bcl-2, Mcl-1L and Bcl-xL levels to 1. At this state, proliferation and self-renewal capacity decreased to 1 and apoptosis increased to 1. However, correct differentiation fluctuated between 0 and 1. This result is illustrated in Figure 3. In BC CML, the same combination of “Imatinib treatment” and inhibitions shows variable efficacy. This variability is broadly consistent with patient response to treatment with Imatinib, where benefits were seen in the short term but long term prognosis remains unsatisfactory^33^. Taken together therefore, comprehensive *in silico* perturbations highlighted the relative stability of CML regulatory states, and also identified several new leads for the development of new therapeutic strategies.

## Discussion

In this study, we constructed and analyzed a genetic model of CML and its progression to the most aggressive blast crisis state using a new biologist-friendly tool for Qualitative Network modeling (BMA). The validity of the model was verified through replicating *in silico* experimental data not utilized in the construction of the model. New combinations of druggable genes were predicted, and a novel mechanistic hypothesis was formed that a fundamental difference may exist between modulating combinations of genes and combinations of genetic interactions. This study not only offers new insight into CML, but has much wider application in the necessary development of suitable data interpretation and modeling tools, which lags behind the corresponding production of complex datasets ubiquitous across virtually all areas of biomedical research. One of the most promising arenas for computational modeling is the field of drug discovery. Because drugs are typically given systemically, reductionist approaches are unsuitable predictors of a drug's effect on the entire system^34^. We elected to develop a network model encapsulating the known biology of CML as the first large real-world application of BMA. This approach had the advantages that (1) CML is genetically tractable because of the uniform initiating translocation event, (2) there is a large body of literature on the genetic pathways relevant for CML biology, and (3) a network model for CML afforded us an opportunity to evaluate the potential utility of modeling using BMA within the context of drug discovery. Importantly, our model showed excellent agreement with known experimental data that were not utilized for its construction. All seven of the experimental results tested that were not used in the construction or refinement of the model essentially agreed with the *in silico* predictions produced. Similar modeling of gene regulatory networks, and indeed even a leukemic model, using Boolean Networks have been performed previously by Saadatpour and Wang in the Albert lab^35^. However, the use of Qualitative Networks and thus the expansion to multiple possible states past the binary restriction of Boolean Networks, as well as ability to integrate multiple inputs and describe more complex relations using target functions explains the greater agreement of model results with experimental data. This degree of accuracy motivated us to perform extensive perturbation analysis which resulted in several noteworthy observations as discussed below.

The lack of detectable cell behavior changes from both the whole gene and gene interaction double KOs was surprising. Though it is perhaps not unexpected that even a genetically relatively “simple” malignancy such as CML will have multiple, at least partially redundant pathways promoting growth and survival, our model of 54 nodes and 104 interactions pared down many pathways to the canonical genes. Therefore, even though the model was complex by comparison to other models in the literature, one would have expected that with such a core set of genes, more genes would be essential for function. Our analysis however suggests that the CML regulatory state is extremely robust, requiring multiple perturbations to elicit changes in cellular behavior that reconfigure CML cellular states towards a more wild type configuration. In some sense, this result confirms the necessity of a systems biology approach, because it seems reasonable to conclude that other cancers which are associated with many more genetic alterations may require even more complex interventions where it would be virtually impossible to make rational predictions on combinatorial perturbations without the aid of advanced modeling approaches.

Another interesting observation providing potential new mechanistic insight is the finding that different genes within the CML regulatory network are critical in cell behavior depending on whether whole genes (network nodes) or just gene-gene interactions (network edges) are removed. The only gene pair showing a disease reversal phenotype in the pairwise network node removal was Bcl-xL, an anti-apoptotic gene, and Ras, a gene upstream in the MAP kinase pathway, yet neither of these genes was present in the gene pairs with phenotype reversal following pairwise network edge removal. Of note, Bcl-xL occupied an endpoint location within the network, and only had one interaction with any other gene in the network. Since removal of the edge to Bcl-xL would be equivalent to removing Bcl-xL altogether, our data highlight the potential to generate different phenotypes with node and edge removals, because none of the other edges, when removed, could generate the same phenotype that was seen following the pairwise edge removal of Bcl-xL and Ras. The potential to generate distinct phenotypes by targeting network nodes and edges enhances the scope for pharmacological intervention significantly, particularly since recent drug development programs have successfully targeted specific protein-protein interactions in addition to the more conventional total inhibitor drugs^36^,^37^. Importantly, the BMA model of CML introduced here provides a platform that facilitates the interrogation of potential phenotypes following combinatorial interventions that can be a complex mixture of drugs that target network nodes and/or edges.

While several of the key genes and genetic interactions found were obvious (such as the removal of Bcr-Abl's repression of its own repressors), others were novel. The pairwise gene KO of IL3R and Bcl-xL for example was unusual in the combination of a broad, upstream and a terminally-downstream gene. Gene interaction analysis also highlighted CrkL as a critical gene in the survival and spread of CML, indicating its significance in Imatinib-resistant CML beyond its current usage as only a predictor of clinical outcome and as a potential druggable target.

We have chosen BMA and Qualitative Networks for the elaboration of our model. BMA's unique feature is that it makes the construction of an initial model extremely simple, allowing users with no prior experience to start work immediately and produce preliminary models^38^,^39^. It supports analysis techniques that are based on formal verification and techniques that were developed in the formal verification community to represent and analyze large state spaces^13^. Thus, BMA offers the ability to analyze very large networks. BMA gives up a priori the attempt to draw the explicit state graph, as this is infeasible even for moderately sized models. At the same time, the interface does allow using the more complex features of Qualitative Networks^8^ and allows an advanced user to create advanced models. We are currently adding more support for additional types of analysis. The successful implementation of BMA in this study highlights its utility as a powerful tool for systems-level computational modeling and subsequent analysis, with applicability across a wide range biomedical research fields.

## Supplementary Material

Supplementary Information Supplementary Information

Supplementary Information Supplementary Tables 1-3

Supplementary Information Supplementary Table 4

Supplementary Information Supplementary Table 5

## Figures and Tables

**Figure 1:**
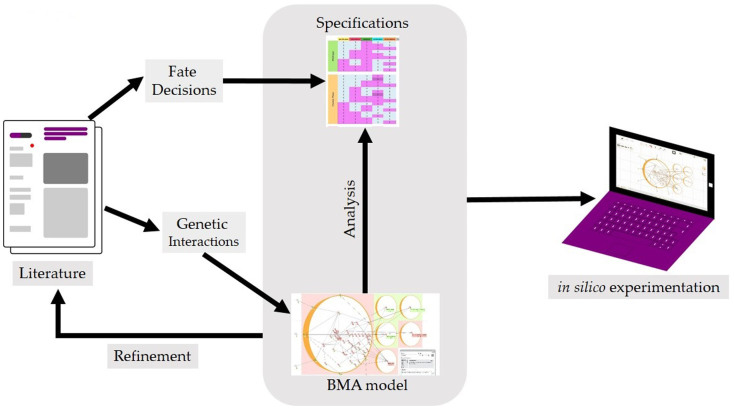
BMA workflow. Genetic interactions curated from the literature are used to build the Qualitative Network in the BMA, whilst the results of known experimental mutations are used to build a “specification” which explicitly links the cell fate to a change in the system. By testing the model in the BMA and comparing the results to the specification, the model is iteratively refined until it matches the specification. Once a working model has been defined, further in silico experiments can be performed to explore new avenues.

**Table 1:** List of experiments verified through the model. Summary of experimental conclusions are given in “Conclusion”, and in silico results are given in “Model Results”

in silico Experimental Details	Conclusion	Source	Model Results
From base CML conditions, set any 2 of the following 3 (RAS, PI3K, or STAT5) to 0. Observe Apoptosis.	2 out of 3 of the following genes need to be active, or else apoptosis occurs in CML cells: RAS, PI3K, or STAT5.	Sonoyama 2002	Apoptosis increases from 0 to 1 with the removal of more than any one of the three genes.
From base CPCML conditions, set all factors (Wnt,VEGF, EPO, IL3, IL6, GH) to 0. From base BCCML conditions, set all factors to 0. Study HPNRK knockout behavior.	HPNRK activation via ERK in BC and CP cell lines supports cytokine independent proliferation, though knock outs reduce cytokine dependent proliferation.	Notari 2006	In the presence of cytokines, both CP and BC CML show reduced proliferation (2 to 1) in the presence of a HPNRK knock out. In the absence of cytokines, knock outs have no effect.
From base CPCML conditions, set constant of Bcr-Abl to 1 instead of 2. Increase EPO to 2. Observe apoptosis.	EPO overcomes apoptosis induced by Imatinib.	Uchida 2004	Apoptosis cycles between 0 and 1 after EPO is raised from 1 to 2.
From base CPCML conditions, set constant of Bcr-Abl to 1 instead of 2. Observe proliferation and apoptosis. From base BCCML conditions, set constant of Bcr-Abl to 1 instead of 2. Observe proliferation and apoptosis.	Imatinib reduces proliferation abnormally upregulated in CML progenitors, and non-specifically induces apoptosis.	Holtz 2002	Reducing Bcr-Abl levels to 1 from 2 in the CPCML and BCCML states lowers proliferation from 2 to 1, and increases apoptosis from 0 to oscillating between 0 and 1.
From base CPCML conditions, set IL3 to 0. Observe proliferation. From base BCCML conditions, set IL3 to 0. Observe proliferation.	IL3 upregulation induces factor independent growth in leukemic cells.	Holyoake 2001	Reducing IL3 from 2 to 0 results in proliferation going from 2 to 1 in CPCML and BCCML states.
From base CPCML conditions, set Bcl-XL to 0. Observe apoptosis. From base CPCML conditions, set Bcl-XL to 1. Observe apoptosis.	Bcl-XL supression induces apoptosis in CML cells.	Oetzel 2000, Horita 2000.	Apoptosis increases from 0 to 1 in both CPCML and BCCML states after reducing Bcl-XL to 0.
From base CPCML conditions, set all factors to 0. Observe proliferation and self-renewal. From base BCCML conditions, set all factors to 0. Observe proliferation and self-renewal.	Bcr-Abl conveys the ability for CML cells to have factor-independent growth	Hariharan 1988	Proliferation and Self-Renewal capacity remain at 1 in CPCML and at 1 and 2 in BCCML even with removal of all factor input from BCCML state.

**Table 2:** Outcomes of double knock-out experiments. Summary of possible outcomes of double knock-out experiments. For each outcome (i.e., levels of growth arrest, self-renewal, apoptosis, proliferation, and differentiation) we list the number of double knock-outs that produce this outcome (repetitions) and one example of such pairwise knock-out

	growth arrest	self renewal	apoptosis	proliferation	differentiation	#repetitions	KO1	KO2
**Wild-type**	0	1	1	0	0	2	CEBPA	cMyc
	1	1	1	0	0	2	cMyc	Fli-1
	0	1	0	1	0	4	Fli-1	FoxO
	0	0	1	1	0	10	Stat3	Fli-1
	0	1	1	1	0	79	CEBPA	TCR
	1	0	1	0	1	21	HNRPK	IL-6 R
	1	1	1	0	1	83	ERK	HNRPK
	0	0	0	1	1	6	FoxO	IL-6 R
	0	1	0	1	1	71	PI3K	Bim/FasL
	0	0	1	1	1	210	IL-6 R	Jun
	0	1	1	1	1	737	JunB	TCR
**Chronic Phase**	0	1	0	0	0	2	cMyc	Jun
	1	1	0	0	0	2	Ras	cMyc
	0	0	0	1	0	8	JAK2	Jun
	0	1	0	1	0	314	IL-6 R	Jun
	1	1	0	1	0	8	MEK	cMyc
	0	0	1	1	0	8	Stat5	CrkL
	0	1	1	1	0	16	IL-3 R	CrkL
	0	0	0	2	0	38	IL-6 R	JAK2
	0	1	0	2	0	702	IL-6 R	TCR
	0	0	1	2	0	42	Stat5	TCR
	0	1	1	2	0	38	Bcl-xL	Frizzled
	1	1	1	0	1	2	Bcr-Abl	cMyc
	0	1	0	1	1	2	Bcr-Abl	FoxO
	0	0	1	1	1	5	Bcr-Abl	IL-6 R
	0	1	1	1	1	38	Bcr-Abl	TCR
**Blast Crisis**	0	2	0	0	0	2	cMyc	Jun
	1	2	0	0	0	2	Ras	cMyc
	0	1	0	1	0	36	Hck	Jun
	0	2	0	1	0	286	JunB	IL-6 R
	1	2	0	1	0	8	Ras	cMyc
	0	1	1	1	0	10	cMyc	Stat5
	0	2	1	1	0	14	IL-3 R	CrkL
	0	0	0	2	0	1	JAK2	BCat
	0	1	0	2	0	154	IL-6 R	Hck
	0	2	0	2	0	585	IL-6 R	TCR
	0	0	1	2	0	1	Stat5	BCat
	0	1	1	2	0	44	Bcl-xL	BCat
	0	2	1	2	0	35	Bcl-xL	IL-6 R
	1	1	1	0	1	2	Bcr-Abl	cMyc
	0	1	0	1	1	2	Bcr-Abl	FoxO
	0	0	1	1	1	5	Bcr-Abl	IL-6 R
	0	1	1	1	1	38	Bcr-Abl	TCR
