# Supplementary material for: Drug Target Optimization in Chronic Myeloid Leukemia Using Innovative Computational Platform
Source: Sci Rep. 2015 Feb 3;5:8190. doi: 10.1038/srep08190 (PMC4650822; doi:10.1038/srep08190)
Supplement: Supplementary Information [file srep08190-s1.doc]

*Scientific Reports – Supplementary Material* 9^th^ December 2014

Supplementary Material

Drug Target Optimization in Chronic Myeloid Leukemia Using Innovative Computational Platform

Ryan Chuang^1,†^, Benjamin A. Hall^2,3,†^, David Benque^2^, Byron Cook^2,4^, Samin Ishtiaq^2^, Nir Piterman^5^, Alex Taylor^2^, Moshe Vardi^6^, Steffen Koschmieder^7^, Berthold Gottgens^8, 9^* and Jasmin Fisher^2, 10^*

^1^ University of Cambridge, Cambridge CB3 0WA, UK

^2^ Microsoft Research, Cambridge CB1 2FB, UK

^3^ MRC Cancer Unit, University of Cambridge, Cambridge, UK CB2 0XZ

^4^ Department of Computer Science, University College London, London, WC1E 6BT, UK;

^5^ Department of Computer Science, University of Leicester, Leicester, LE1 7RH, UK

^6^ Department of Computer Science, Rice University, Huston, 77005-1892, Texas

^7^ Department of Medicine, University Hospital of Aachen, Aachen, D-52074, Germany

^8^ Cambridge Institute for Medical Research, University of Cambridge, Cambridge CB2 0XY, UK

^9^ Wellcome Trust and MRC Cambridge Stem Cell Institute, Cambridge CB2 0XY, UK

^10^ Department of Biochemistry, University of Cambridge, Cambridge CB2 1GA, UK

*Correspondence should be addressed to [jasmin.fisher@microsoft.com](mailto:jasmin.fisher@microsoft.com) or [jf416@cam.ac.uk](mailto:jf416@cam.ac.uk) and [bg200@cam.ac.uk](mailto:bg200@cam.ac.uk)

**List of Supplementary Figures and Tables**

Supplementary Figure S1.

Supplementary Figure S2

All Supplementary tables are available at: [**http://www.cs.le.ac.uk/people/np183/publications/2014/CML/**](http://www.cs.le.ac.uk/people/np183/publications/2014/CML/)

Supplementary Table S1. (Tab 1 in file SupplementaryTables1-3.xlsx)

Supplementary Table S2. (Tab 2 in file SupplementaryTables1-3.xlsx)

Supplementary Table S3. (Tab 3 in file SupplementaryTables1-3.xlsx)

Supplementary Table S4. (4 Tabs in file SupplementaryTable4.xlsx)

Supplementary Table S5. (6 Tabs in file SupplementaryTable5.xlsx)

**Supplementary Figure and Table Legends**

## Figure S1. Illustration of model building through the BioModelAnalyzer Graphic User Interface (BMA GUI). (A) Membrane bound receptors are depicted in green, intracellular stand-alone proteins are depicted in red, constant proteins and cell factors are depicted in grey, and interactions are depicted as either pointed (positive) or bar (negative) arrows. Reciprocal interactions, or two-directional edges, are supported (not depicted). (B) Several input proteins (b-e) influencing the value of a single protein (a).

**Figure S2. Stabilization analysis of the result found by Holtz et al. 2002 summarized in Table 1.** **(A)** Base CP CML Model. (**B)** CP CML model after reducing the Bcr-Abl constant from 2 to 1. Note that in addition to the stable result of the proliferation phenotype shifting from 2 to 1, Apoptosis and Correct Differentiation shift from the stable values of 0 to oscillating between values 0 and 1, leading the entirety of the model to be labeled as a failure to reach stability.

**Table S1.** **List of nodes in the network.** Network ID refers to the internal label of the node. Proper name is Entrez name while Network Name is name given in construction of model. Cell fate is given for terminally‐downstream gene nodes.

## Table S2. List of edges in the network. BMA ID, Source ID, and Target ID refer to internal label of the node.

## Table S3. List of edges in the network and corresponding sources.

## Table S4.

***1^st^ Subtable:***

KO1 and KO2 are the nodes knocked out identified by internal BMA ID as labeled in Supplementary Table 1 under the heading "Network ID." GA, SRC, A, P, CD are resulting phenotypes for the model - Growth Arrest, Self-Renewal Capacity, Apoptosis, Proliferation, and Correct Differentiation. A value of 0 corresponds to an extremely low or not present state of the phenotype and is labeled in red, a value of 1 is a moderate state and is labeled in yellow, and a value of 2 is a high state and is labeled in green.

***2^nd^ Subtable:***

Columns represent model results where double KOs in each of the three base models (Wild Type (WT), Chronic Phase (CP) and Blast Crisis (BC)) resulted in the same values for the six phenotype nodes. A value of 1 corresponds to the same values and is labeled in green. A value of 0 corresponds to dissimilar values and is labeled in red.

***3^rd^ Subtable:***

Columns represent model results where double KOs in each of the three base models resulted in a normal phenotype (corresponding to values of 0 for growth arrest, and 1 for each of the other phenotype nodes.) A value of 1 corresponds to when this occurs and is labeled in green. A value of 0 corresponds to when this does not occur and is labeled in red.

***4^th^ Subtable:***

Columns represent model results where double KOs in each of the three base models resulted in a normal phenotype (as described in the "Third Subtable") for the WT model, and where CP and BC models showed reduced survival and proliferation, defined as when SRC is below 2, Apoptosis is above 0, Proliferation is below 2, and Correct Differentiation is below 2. A value of 1 corresponds to when this occurs and is labeled in green. A value of 0 corresponds to when this does not occur and is labeled in red.

## Table S5.

KO1 and KO2 are the nodes knocked out identified by internal BMA ID as labeled in Supplementary Table 2 under the heading "BMA ID." Please see the above legend for detailed description of each of the subtables.

**Supplementary Figures**


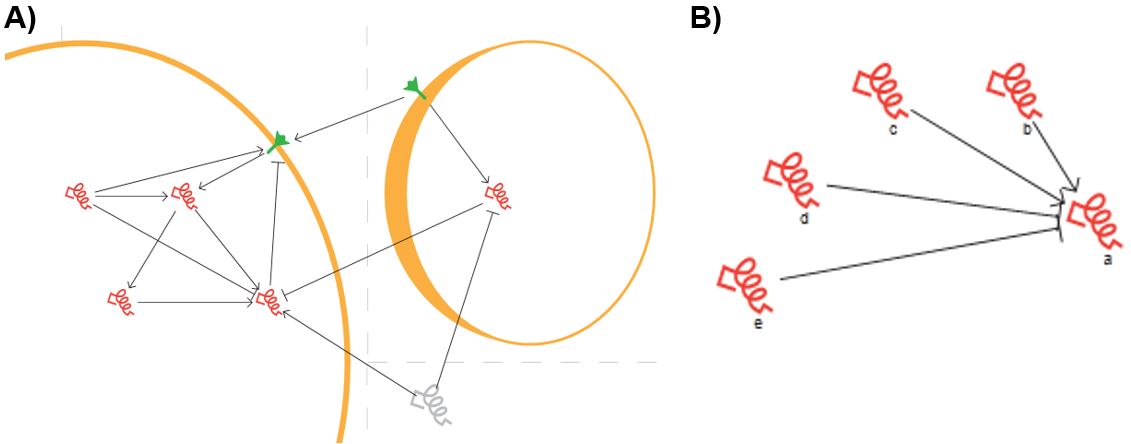


## Figure S1. Illustration of model building through the BioModelAnalyzer Graphic User Interface (BMA GUI). (A) Membrane bound receptors are depicted in green, intracellular stand-alone proteins are depicted in red, constant proteins and cell factors are depicted in grey, and interactions are depicted as either pointed (positive) or bar (negative) arrows. Reciprocal interactions, or two-directional edges, are supported (not depicted). (B) Several input proteins (b-e) influencing the value of a single protein (a).

**
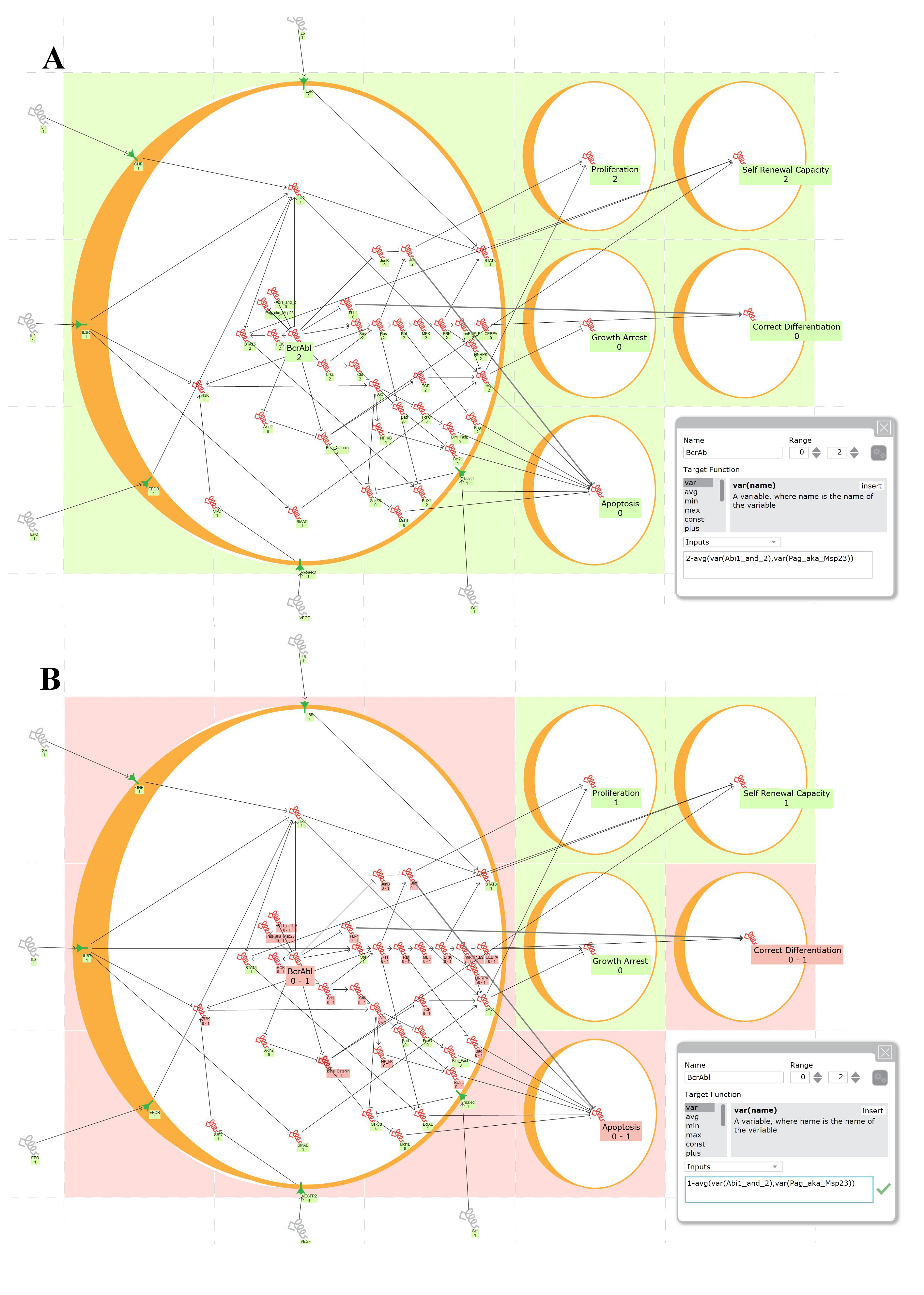
**

**Figure S2. Stabilization analysis of the result found by Holtz et al. 2002 summarized in Table 1.** **(A)** Base CP CML Model. (**B)** CP CML model after reducing the Bcr-Abl constant from 2 to 1. Note that in addition to the stable result of the proliferation phenotype shifting from 2 to 1, Apoptosis and Correct Differentiation shift from the stable values of 0 to oscillating between values 0 and 1, leading the entirety of the model to be labeled as a failure to reach stability.

**Supplementary References**

1. K. Antoku, R. S. Maser, W. J. Scully, S. M. Delach, D. E. Johnson, Isolation of Bcl-2 binding proteins that exhibit homology with BAG-1 and suppressor of death domains protein. Biochem Biophys Res Commun 286, 1003-1010 (2001).
2. D. S. Askew, R. A. Ashmun, B. C. Simmons, J. L. Cleveland, Constitutive c-myc expression in an IL-3-dependent myeloid cell line suppresses cell cycle arrest and accelerates apoptosis. Oncogene 6, 1915-1922 (1991).
3. J. Bachmann, A. Raue, M. Schilling, M. E. Bohm, C. Kreutz, D. Kaschek, H. Busch, N. Gretz, W. D. Lehmann, J. Timmer, U. Klingmuller, Division of labor by dual feedback regulators controls JAK2/STAT5 signaling over broad ligand range. Mol Syst Biol 7, 516-516 (2011).
4. J. L. Barclay, S. T. Anderson, M. J. Waters, J. D. Curlewis, Characterization of the SOCS3 promoter response to prostaglandin E2 in T47D cells. Mol Endocrinol 21, 2516-2528 (2007).
5. F. Belloc, F. Moreau-Gaudry, M. Uhalde, L. Cazalis, M. Jeanneteau, F. Lacombe, V. Praloran, F.-X. Mahon, Imatinib and nilotinib induce apoptosis of chronic myeloid leukemia cells through a Bim-dependant pathway modulated by cytokines. Cancer Biol Ther 6, 912-919 (2007).
6. T. P. Beyer A, Li X, Scott J, Fisher J, Mechanistic Insights into Metabolic Disturbance during Type-II Diabetes and Obesity using Qualitative Networks. . Transactions on Computational Systems Biology, 146-162 (2010).
7. P. Bhanot, M. Brink, C. H. Samos, J. C. Hsieh, Y. Wang, J. P. Macke, D. Andrew, J. Nathans, R. Nusse, A new member of the frizzled family from Drosophila functions as a Wingless receptor. Nature 382, 225-230 (1996).
8. R. P. Bissonnette, F. Echeverri, A. Mahboubi, D. R. Green, Apoptotic cell death induced by c-myc is inhibited by bcl-2. Nature 359, 552-554 (1992).
9. W. M. Blankesteijn, V. A. M. van de Schans, P. ter Horst, J. F. M. Smits, The Wnt/frizzled/GSK-3 beta pathway: a novel therapeutic target for cardiac hypertrophy. Trends Pharmacol Sci 29, 175-180 (2008).
10. E. Bossy-Wetzel, L. Bakiri, M. Yaniv, Induction of apoptosis by the transcription factor c-Jun. EMBO J 16, 1695-1709 (1997).
11. A. Brunet, A. Bonni, M. J. Zigmond, M. Z. Lin, P. Juo, L. S. Hu, M. J. Anderson, K. C. Arden, J. Blenis, M. E. Greenberg, Akt promotes cell survival by phosphorylating and inhibiting a Forkhead transcription factor. Cell 96, 857-868 (1999).
12. D. Buet, H. Raslova, J. F. Geay, P. Jarrier, V. Lazar, A. Turhan, F. Morle, W. Vainchenker, F. Louache, p210(BCR-ABL) reprograms transformed and normal human megakaryocytic progenitor cells into erythroid cells and suppresses FLI-1 transcription. Leukemia 21, 917-925 (2007).
13. G. J. Buttrick, J. G. Wakefield, PI3-K and GSK-3: Akt-ing together with microtubules. Cell Cycle 7, 2621-2625 (2008).
14. B. Calabretta, D. Perrotti, The biology of CML blast crisis. Blood 103, 4010-4022 (2004).
15. N. Carlesso, D. A. Frank, J. D. Griffin, Tyrosyl phosphorylation and DNA binding activity of signal transducers and activators of transcription (STAT) proteins in hematopoietic cell lines transformed by Bcr/Abl. J Exp Med 183, 811-820 (1996).
16. E. Caron, S. Ghosh, Y. Matsuoka, D. Ashton-Beaucage, M. Therrien, S. Lemieux, C. Perreault, P. P. Roux, H. Kitano, A comprehensive map of the mTOR signaling network. Mol Syst Biol 6, 453 (2010).
17. S. K. Chai, G. L. Nichols, P. Rothman, Constitutive activation of JAKs and STATs in BCR-Abl-expressing cell lines and peripheral blood cells derived from leukemic patients. J Immunol 159, 4720-4728 (1997).
18. F. Chang, L. S. Steelman, J. T. Lee, J. G. Shelton, P. M. Navolanic, W. L. Blalock, R. A. Franklin, J. A. McCubrey, Signal transduction mediated by the Ras/Raf/MEK/ERK pathway from cytokine receptors to transcription factors: potential targeting for therapeutic intervention. Leukemia 17, 1263-1293 (2003).
19. J. S. Chang, R. Santhanam, R. Trotta, P. Neviani, A. M. Eiring, E. Briercheck, M. Ronchetti, D. C. Roy, B. Calabretta, M. A. Caligiuri, D. Perrotti, High levels of the BCR/ABL oncoprotein are required for the MAPK-hnRNP-E2 dependent suppression of C/EBPalpha-driven myeloid differentiation. Blood 110, 994-991003 (2007).
20. L. Chang, M. Karin, Mammalian MAP kinase signalling cascades. Nature 410, 37-40 (2001).
21. D. T. Chao, G. P. Linette, L. H. Boise, L. S. White, C. B. Thompson, S. J. Korsmeyer, Bcl-XL and Bcl-2 repress a common pathway of cell death. J Exp Med 182, 821-828 (1995).
22. T. Cheng, H. Shen, D. Giokas, J. Gere, D. G. Tenen, D. T. Scadden, Temporal mapping of gene expression levels during the differentiation of individual primary hematopoietic cells. Proc Natl Acad Sci U S A 93, 13158-13163 (1996).
23. H. Cheon, Y. H. Rho, S. J. Choi, Y. H. Lee, G. G. Song, J. Sohn, N. H. Won, J. D. Ji, Prostaglandin E2 augments IL-10 signaling and function. J Immunol 177, 1092-1100 (2006).
24. J. C. Chow, P. R. Ling, Z. Qu, L. Laviola, A. Ciccarone, B. R. Bistrian, R. J. Smith, Growth hormone stimulates tyrosine phosphorylation of JAK2 and STAT5, but not insulin receptor substrate-1 or SHC proteins in liver and skeletal muscle of normal rats in vivo. Endocrinology 137, 2880-2886 (1996).
25. J. Chung, E. Uchida, T. C. Grammer, J. Blenis, STAT3 serine phosphorylation by ERK-dependent and -independent pathways negatively modulates its tyrosine phosphorylation. Mol Cell Biol 17, 6508-6516 (1997).
26. M. H. Cobb, MAP kinase pathways. Prog Biophys Mol Biol 71, 479-500 (1999).
27. A. M. L. Coluccia, A. Vacca, M. Dunach, L. Mologni, S. Redaelli, V. H. Bustos, D. Benati, L. A. Pinna, C. Gambacorti-Passerini, Bcr-Abl stabilizes beta-catenin in chronic myeloid leukemia through its tyrosine phosphorylation. EMBO J 26, 1456-1466 (2007).
28. B. Cook, J. Fisher, E. Krepska, N. Piterman, Proving Stabilization of Biological Systems. Verification, Model Checking, and Abstract Interpretation 6538, 134-149 (2011).
29. S. Cory, D. C. S. Huang, J. M. Adams, The Bcl-2 family: roles in cell survival and oncogenesis. Oncogene 22, 8590-8607 (2003).
30. B. L. Craddock, M. J. Welham, Interleukin-3 induces association of the protein-tyrosine phosphatase SHP2 and phosphatidylinositol 3-kinase with a 100-kDa tyrosine-phosphorylated protein in hemopoietic cells. J Biol Chem 272, 29281-29289 (1997).
31. B. C. Cunningham, M. Ultsch, A. M. De Vos, M. G. Mulkerrin, K. R. Clauser, J. A. Wells, Dimerization of the extracellular domain of the human growth hormone receptor by a single hormone molecule. Science 254, 821-825 (1991).
32. Z. Dai, R. C. Quackenbush, K. D. Courtney, M. Grove, D. Cortez, G. W. Reuther, A. M. Pendergast, Oncogenic Abl and Src tyrosine kinases elicit the ubiquitin-dependent degradation of target proteins through a Ras-independent pathway. Genes Dev 12, 1415-1424 (1998).
33. A. D. D'Andrea, H. F. Lodish, G. G. Wong, Expression cloning of the murine erythropoietin receptor. Cell 57, 277-285 (1989).
34. R. P. de Groot, J. A. Raaijmakers, J. W. Lammers, R. Jove, L. Koenderman, STAT5 activation by BCR-Abl contributes to transformation of K562 leukemia cells. Blood 94, 1108-1112 (1999).
35. B. J. Druker, F. Guilhot, S. G. O'Brien, I. Gathmann, H. Kantarjian, N. Gattermann, M. W. Deininger, R. T. Silver, J. M. Goldman, R. M. Stone, F. Cervantes, A. Hochhaus, B. L. Powell, J. L. Gabrilove, P. Rousselot, J. Reiffers, J. J. Cornelissen, T. Hughes, H. Agis, T. Fischer, G. Verhoef, J. Shepherd, G. Saglio, A. Gratwohl, J. L. Nielsen, J. P. Radich, B. Simonsson, K. Taylor, M. Baccarani, C. So, L. Letvak, R. A. Larson, I. Investigators, Five-year follow-up of patients receiving imatinib for chronic myeloid leukemia. N Engl J Med 355, 2408-2417 (2006).
36. B. J. Druker, C. L. Sawyers, H. Kantarjian, D. J. Resta, S. F. Reese, J. M. Ford, R. Capdeville, M. Talpaz, Activity of a specific inhibitor of the BCR-ABL tyrosine kinase in the blast crisis of chronic myeloid leukemia and acute lymphoblastic leukemia with the Philadelphia chromosome. N Engl J Med 344, 1038-1042 (2001).
37. M. Eilers, R. N. Eisenman, Myc's broad reach. Genes Dev 22, 2755-2766 (2008).
38. B. P. Eliceiri, R. Paul, P. L. Schwartzberg, J. D. Hood, J. Leng, D. A. Cheresh, Selective requirement for Src kinases during VEGF-induced angiogenesis and vascular permeability. Mol Cell 4, 915-924 (1999).
39. B. Emanuelli, P. Peraldi, C. Filloux, D. Sawka-Verhelle, D. Hilton, E. Van Obberghen, SOCS-3 is an insulin-induced negative regulator of insulin signaling. J Biol Chem 275, 15985-15991 (2000).
40. G. I. Evan, A. H. Wyllie, C. S. Gilbert, T. D. Littlewood, H. Land, M. Brooks, C. M. Waters, L. Z. Penn, D. C. Hancock, Induction of apoptosis in fibroblasts by c-myc protein. Cell 69, 119-128 (1992).
41. X. Fang, S. Yu, A. Eder, M. Mao, R. C. Bast, D. Boyd, G. B. Mills, Regulation of BAD phosphorylation at serine 112 by the Ras-mitogen-activated protein kinase pathway. Oncogene 18, 6635-6640 (1999).
42. A. Fanidi, E. A. Harrington, G. I. Evan, Cooperative interaction between c-myc and bcl-2 proto-oncogenes. Nature 359, 554-556 (1992).
43. S. M. Feller, B. Knudsen, H. Hanafusa, c-Abl kinase regulates the protein binding activity of c-Crk. EMBO J 13, 2341-2351 (1994).
44. X.-H. Feng, Y.-Y. Liang, M. Liang, W. Zhai, X. Lin, Direct interaction of c-Myc with Smad2 and Smad3 to inhibit TGF-beta-mediated induction of the CDK inhibitor p15(Ink4B). Mol Cell 9, 133-143 (2002).
45. C. R. Geest, M. Buitenhuis, A. G. Laarhoven, M. B. Bierings, M. C. A. Bruin, E. Vellenga, P. J. Coffer, p38 MAP kinase inhibits neutrophil development through phosphorylation of C/EBPalpha on serine 21. Stem Cells 27, 2271-2282 (2009).
46. S. Ghaffari, Z. Jagani, C. Kitidis, H. F. Lodish, R. Khosravi-Far, Cytokines and BCR-ABL mediate suppression of TRAIL-induced apoptosis through inhibition of forkhead FOXO3a transcription factor. Proc Natl Acad Sci U S A 100, 6523-6528 (2003).
47. A. Gouble, D. Morello, Synchronous and regulated expression of two AU-binding proteins, AUF1 and HuR, throughout murine development. Oncogene 19, 5377-5384 (2000).
48. D. Hanahan, R. A. Weinberg, The hallmarks of cancer. Cell 100, 57-70 (2000).
49. O. Hantschel, G. Superti-Furga, Regulation of the c-Abl and Bcr-Abl tyrosine kinases. Nat Rev Mol Cell Biol 5, 33-44 (2004).
50. O. Hantschel, W. Warsch, E. Eckelhart, I. Kaupe, F. Grebien, K.-U. Wagner, G. Superti-Furga, V. Sexl, BCR-ABL uncouples canonical JAK2-STAT5 signaling in chronic myeloid leukemia. Nat Chem Biol 8, 285-293 (2012).
51. S. Herold, M. Wanzel, V. Beuger, C. Frohme, D. Beul, T. Hillukkala, J. Syvaoja, H.-P. Saluz, F. Haenel, M. Eilers, Negative regulation of the mammalian UV response by Myc through association with Miz-1. Mol Cell 10, 509-521 (2002).
52. M. Hibi, M. Murakami, M. Saito, T. Hirano, T. Taga, T. Kishimoto, Molecular cloning and expression of an IL-6 signal transducer, gp130. Cell 63, 1149-1157 (1990).
53. K. Holmes, O. L. Roberts, A. M. Thomas, M. J. Cross, Vascular endothelial growth factor receptor-2: structure, function, intracellular signalling and therapeutic inhibition. Cell Signal 19, 2003-2012 (2007).
54. M. Horita, E. J. Andreu, A. Benito, C. Arbona, C. Sanz, I. Benet, F. Prosper, J. L. Fernandez-Luna, Blockade of the Bcr-Abl kinase activity induces apoptosis of chronic myelogenous leukemia cells by suppressing signal transducer and activator of transcription 5-dependent expression of Bcl-xL. J Exp Med 191, 977-984 (2000).
55. S. Y. Hsu, A. Kaipia, L. Zhu, A. J. Hsueh, Interference of BAD (Bcl-xL/Bcl-2-associated death promoter)-induced apoptosis in mammalian cells by 14-3-3 isoforms and P11. Mol Endocrinol 11, 1858-1867 (1997).
56. D. C. Huang, M. Hahne, M. Schroeter, K. Frei, A. Fontana, A. Villunger, K. Newton, J. Tschopp, A. Strasser, Activation of Fas by FasL induces apoptosis by a mechanism that cannot be blocked by Bcl-2 or Bcl-x(L). Proc Natl Acad Sci U S A 96, 14871-14876 (1999).
57. M. Hucka, A. Finney, H. M. Sauro, H. Bolouri, J. C. Doyle, H. Kitano, A. P. Arkin, B. J. Bornstein, D. Bray, A. Cornish-Bowden, A. A. Cuellar, S. Dronov, E. D. Gilles, M. Ginkel, V. Gor, Goryanin, II, W. J. Hedley, T. C. Hodgman, J. H. Hofmeyr, P. J. Hunter, N. S. Juty, J. L. Kasberger, A. Kremling, U. Kummer, N. Le Novere, L. M. Loew, D. Lucio, P. Mendes, E. Minch, E. D. Mjolsness, Y. Nakayama, M. R. Nelson, P. F. Nielsen, T. Sakurada, J. C. Schaff, B. E. Shapiro, T. S. Shimizu, H. D. Spence, J. Stelling, K. Takahashi, M. Tomita, J. Wagner, J. Wang, S. Forum, The systems biology markup language (SBML): a medium for representation and exchange of biochemical network models. Bioinformatics 19, 524-531 (2003).
58. T. P. Hughes, J. Kaeda, S. Branford, Z. Rudzki, A. Hochhaus, M. L. Hensley, I. Gathmann, A. E. Bolton, I. C. van Hoomissen, J. M. Goldman, J. P. Radich, S. T. I. S. G. International Randomised Study of Interferon versus, Frequency of major molecular responses to imatinib or interferon alfa plus cytarabine in newly diagnosed chronic myeloid leukemia. N Engl J Med 349, 1423-1432 (2003).
59. T. Ideker, T. Galitski, L. Hood, A new approach to decoding life: systems biology. Annu Rev Genomics Hum Genet 2, 343-372 (2001).
60. A. Ikeda, M. Ueguchi-Tanaka, Y. Sonoda, H. Kitano, M. Koshioka, Y. Futsuhara, M. Matsuoka, J. Yamaguchi, slender rice, a constitutive gibberellin response mutant, is caused by a null mutation of the SLR1 gene, an ortholog of the height-regulating gene GAI/RGA/RHT/D8. Plant Cell 13, 999-1010 (2001).
61. H. Itoh, M. Ueguchi-Tanaka, N. Sentoku, H. Kitano, M. Matsuoka, M. Kobayashi, Cloning and functional analysis of two gibberellin 3 beta -hydroxylase genes that are differently expressed during the growth of rice. Proc Natl Acad Sci U S A 98, 8909-8914 (2001).
62. E. Jabbour, S. El Ahdab, J. Cortes, H. Kantarjian, Nilotinib: a novel Bcr-Abl tyrosine kinase inhibitor for the treatment of leukemias. Expert Opin Investig Drugs 17, 1127-1136 (2008).
63. C. H. Jamieson, Chronic myeloid leukemia stem cells. Hematology Am Soc Hematol Educ Program, 436-442 (2008).
64. C. H. M. Jamieson, L. E. Ailles, S. J. Dylla, M. Muijtjens, C. Jones, J. L. Zehnder, J. Gotlib, K. Li, M. G. Manz, A. Keating, C. L. Sawyers, I. L. Weissman, Granulocyte-macrophage progenitors as candidate leukemic stem cells in blast-crisis CML. N Engl J Med 351, 657-667 (2004).
65. C. H. M. Jamieson, I. L. Weissman, E. Passegue, Chronic versus acute myelogenous leukemia: a question of self-renewal. Cancer Cell 6, 531-533 (2004).
66. M. Kamio, T. Yoshida, H. Ogata, T. Douchi, Y. Nagata, M. Inoue, M. Hasegawa, Y. Yonemitsu, A. Yoshimura, SOCS1 [corrected] inhibits HPV-E7-mediated transformation by inducing degradation of E7 protein. Oncogene 23, 3107-3115 (2004).
67. Y. Kato, A. Iwama, Y. Tadokoro, K. Shimoda, M. Minoguchi, S. Akira, M. Tanaka, A. Miyajima, T. Kitamura, H. Nakauchi, Selective activation of STAT5 unveils its role in stem cell self-renewal in normal and leukemic hematopoiesis. J Exp Med 202, 169-179 (2005).
68. R. Ketteler, C. S. Moghraby, J. G. Hsiao, O. Sandra, H. F. Lodish, U. Klingmuller, The cytokine-inducible Scr homology domain-containing protein negatively regulates signaling by promoting apoptosis in erythroid progenitor cells. J Biol Chem 278, 2654-2660 (2003).
69. M. Kieslinger, I. Woldman, R. Moriggl, J. Hofmann, J. C. Marine, J. N. Ihle, H. Beug, T. Decker, Antiapoptotic activity of Stat5 required during terminal stages of myeloid differentiation. Genes Dev 14, 232-244 (2000).
70. A. Klejman, S. J. Schreiner, M. Nieborowska-Skorska, A. Slupianek, M. Wilson, T. E. Smithgall, T. Skorski, The Src family kinase Hck couples BCR/ABL to STAT5 activation in myeloid leukemia cells. EMBO J 21, 5766-5774 (2002).
71. P. S. Knoepfler, P. F. Cheng, R. N. Eisenman, N-myc is essential during neurogenesis for the rapid expansion of progenitor cell populations and the inhibition of neuronal differentiation. Genes Dev 16, 2699-2712 (2002).
72. N. Kobayashi, K. Saeki, A. Yuo, Granulocyte-macrophage colony-stimulating factor and interleukin-3 induce cell cycle progression through the synthesis of c-Myc protein by internal ribosome entry site-mediated translation via phosphatidylinositol 3-kinase pathway in human factor-dependent leukemic cells. Blood 102, 3186-3195 (2003).
73. T. Kodaki, R. Woscholski, B. Hallberg, P. Rodriguez-Viciana, J. Downward, P. J. Parker, The activation of phosphatidylinositol 3-kinase by Ras. Curr Biol 4, 798-806 (1994).
74. W. Kolch, Meaningful relationships: the regulation of the Ras/Raf/MEK/ERK pathway by protein interactions. Biochem J 351 Pt 2, 289-305 (2000).
75. F. T. Kolligs, G. Hu, C. V. Dang, E. R. Fearon, Neoplastic transformation of RK3E by mutant beta-catenin requires deregulation of Tcf/Lef transcription but not activation of c-myc expression. Mol Cell Biol 19, 5696-5706 (1999).
76. S. Koschmieder, W. K. Hofmann, J. Kunert, S. Wagner, K. Ballas, G. Seipelt, D. Hoelzer, O. G. Ottmann, U. Kalina, TGF beta-induced SMAD2 phosphorylation predicts inhibition of thymidine incorporation in CD34+ cells from healthy donors, but not from patients with AML after MDS. Leukemia 15, 942-949 (2001).
77. K. M. Kozopas, T. Yang, H. L. Buchan, P. Zhou, R. W. Craig, MCL1, a gene expressed in programmed myeloid cell differentiation, has sequence similarity to BCL2. Proc Natl Acad Sci U S A 90, 3516-3520 (1993).
78. T. S. Lewis, P. S. Shapiro, N. G. Ahn, Signal transduction through MAP kinase cascades. Adv Cancer Res 74, 49-4139 (1998).
79. M. N. Lioubin, P. A. Algate, S. Tsai, K. Carlberg, A. Aebersold, L. R. Rohrschneider, p150Ship, a signal transduction molecule with inositol polyphosphate-5-phosphatase activity. Genes Dev 10, 1084-1095 (1996).
80. T. Liu, A. J. DeCostanzo, X. Liu, H. Wang, S. Hallagan, R. T. Moon, C. C. Malbon, G protein signaling from activated rat frizzled-1 to the beta-catenin-Lef-Tcf pathway. Science 292, 1718-1722 (2001).
81. X. Liu, L. E. Marengere, C. A. Koch, T. Pawson, The v-Src SH3 domain binds phosphatidylinositol 3'-kinase. Mol Cell Biol 13, 5225-5232 (1993).
82. B. T. MacDonald, K. Tamai, X. He, Wnt/beta-catenin signaling: components, mechanisms, and diseases. Dev Cell 17, 9-26 (2009).
83. K. Makanae, R. Kintaka, T. Makino, H. Kitano, H. Moriya, Identification of dosage-sensitive genes in Saccharomyces cerevisiae using the genetic tug-of-war method. Genome Res 23, 300-311 (2013).
84. R. Marais, Y. Light, H. F. Paterson, C. J. Marshall, Ras recruits Raf-1 to the plasma membrane for activation by tyrosine phosphorylation. EMBO J 14, 3136-3145 (1995).
85. B. Markova, C. Albers, F. Breitenbuecher, J. V. Melo, T. H. Brummendorf, F. Heidel, D. Lipka, J. Duyster, C. Huber, T. Fischer, Novel pathway in Bcr-Abl signal transduction involves Akt-independent, PLC-gamma1-driven activation of mTOR/p70S6-kinase pathway. Oncogene 29, 739-751 (2010).
86. A. Matsumoto, M. Masuhara, K. Mitsui, M. Yokouchi, M. Ohtsubo, H. Misawa, A. Miyajima, A. Yoshimura, CIS, a cytokine inducible SH2 protein, is a target of the JAK-STAT5 pathway and modulates STAT5 activation. Blood 89, 3148-3154 (1997).
87. J. V. Melo, D. J. Barnes, Chronic myeloid leukaemia as a model of disease evolution in human cancer. Nat Rev Cancer 7, 441-453 (2007).
88. J. R. Miller, A. M. Hocking, J. D. Brown, R. T. Moon, Mechanism and function of signal transduction by the Wnt/beta-catenin and Wnt/Ca2+ pathways. Oncogene 18, 7860-7872 (1999).
89. O. Miura, Y. Miura, N. Nakamura, F. W. Quelle, B. A. Witthuhn, J. N. Ihle, N. Aoki, Induction of tyrosine phosphorylation of Vav and expression of Pim-1 correlates with Jak2-mediated growth signaling from the erythropoietin receptor. Blood 84, 4135-4141 (1994).
90. R. Monni, S. C. Santos, M. Mauchauffe, R. Berger, J. Ghysdael, F. Gouilleux, S. Gisselbrecht, O. Bernard, V. Penard-Lacronique, The TEL-Jak2 oncoprotein induces Socs1 expression and altered cytokine response in Ba/F3 cells. Oncogene 20, 849-858 (2001).
91. A. L. Mui, H. Wakao, A. M. O'Farrell, N. Harada, A. Miyajima, Interleukin-3, granulocyte-macrophage colony stimulating factor and interleukin-5 transduce signals through two STAT5 homologs. EMBO J 14, 1166-1175 (1995).
92. T. Nakamura, F. Hamada, T. Ishidate, K. Anai, K. Kawahara, K. Toyoshima, T. Akiyama, Axin, an inhibitor of the Wnt signalling pathway, interacts with beta-catenin, GSK-3beta and APC and reduces the beta-catenin level. Genes Cells 3, 395-403 (1998).
93. C. Nerlov, Targeting a chronic problem: elimination of cancer stem cells in CML. EMBO J 28, 167-168 (2009).
94. G. L. Nichols, M. A. Raines, J. C. Vera, L. Lacomis, P. Tempst, D. W. Golde, Identification of CRKL as the constitutively phosphorylated 39-kD tyrosine phosphoprotein in chronic myelogenous leukemia cells. Blood 84, 2912-2918 (1994).
95. H. Niwa, T. Burdon, I. Chambers, A. Smith, Self-renewal of pluripotent embryonic stem cells is mediated via activation of STAT3. Genes Dev 12, 2048-2060 (1998).
96. A. J. Obaya, I. Kotenko, M. D. Cole, J. M. Sedivy, The proto-oncogene c-myc acts through the cyclin-dependent kinase (Cdk) inhibitor p27(Kip1) to facilitate the activation of Cdk4/6 and early G(1) phase progression. J Biol Chem 277, 31263-31269 (2002).
97. T. Oda, C. Heaney, J. R. Hagopian, K. Okuda, J. D. Griffin, B. J. Druker, Crkl is the major tyrosine-phosphorylated protein in neutrophils from patients with chronic myelogenous leukemia. J Biol Chem 269, 22925-22928 (1994).
98. T. Oskarsson, M. A. G. Essers, N. Dubois, S. Offner, C. Dubey, C. Roger, D. Metzger, P. Chambon, E. Hummler, P. Beard, A. Trumpp, Skin epidermis lacking the c-Myc gene is resistant to Ras-driven tumorigenesis but can reacquire sensitivity upon additional loss of the p21Cip1 gene. Genes Dev 20, 2024-2029 (2006).
99. E. Passegue, W. Jochum, M. Schorpp-Kistner, U. Mohle-Steinlein, E. F. Wagner, Chronic myeloid leukemia with increased granulocyte progenitors in mice lacking junB expression in the myeloid lineage. Cell 104, 21-32 (2001).
100. E. Passegue, E. F. Wagner, I. L. Weissman, JunB deficiency leads to a myeloproliferative disorder arising from hematopoietic stem cells. Cell 119, 431-443 (2004).
101. M. Peifer, L. M. Pai, M. Casey, Phosphorylation of the Drosophila adherens junction protein Armadillo: roles for wingless signal and zeste-white 3 kinase. Dev Biol 166, 543-556 (1994).
102. A. M. Pendergast, L. A. Quilliam, L. D. Cripe, C. H. Bassing, Z. Dai, N. Li, A. Batzer, K. M. Rabun, C. J. Der, J. Schlessinger, BCR-ABL-induced oncogenesis is mediated by direct interaction with the SH2 domain of the GRB-2 adaptor protein. Cell 75, 175-185 (1993).
103. D. Perrotti, V. Cesi, R. Trotta, C. Guerzoni, G. Santilli, K. Campbell, A. Iervolino, F. Condorelli, C. Gambacorti-Passerini, M. A. Caligiuri, B. Calabretta, BCR-ABL suppresses C/EBPalpha expression through inhibitory action of hnRNP E2. Nat Genet 30, 48-58 (2002).
104. L. Puil, J. Liu, G. Gish, G. Mbamalu, D. Bowtell, P. G. Pelicci, R. Arlinghaus, T. Pawson, Bcr-Abl oncoproteins bind directly to activators of the Ras signalling pathway. EMBO J 13, 764-773 (1994).
105. F. W. Quelle, N. Sato, B. A. Witthuhn, R. C. Inhorn, M. Eder, A. Miyajima, J. D. Griffin, J. N. Ihle, JAK2 associates with the beta c chain of the receptor for granulocyte-macrophage colony-stimulating factor, and its activation requires the membrane-proximal region. Mol Cell Biol 14, 4335-4341 (1994).
106. A. Quintas-Cardama, H. Kantarjian, D. Jones, J. Shan, G. Borthakur, D. Thomas, S. Kornblau, S. O'Brien, J. Cortes, Delayed achievement of cytogenetic and molecular response is associated with increased risk of progression among patients with chronic myeloid leukemia in early chronic phase receiving high-dose or standard-dose imatinib therapy. Blood 113, 6315-6321 (2009).
107. J. P. Radich, H. Dai, M. Mao, V. Oehler, J. Schelter, B. Druker, C. Sawyers, N. Shah, W. Stock, C. L. Willman, S. Friend, P. S. Linsley, Gene expression changes associated with progression and response in chronic myeloid leukemia. Proc Natl Acad Sci U S A 103, 2794-2799 (2006).
108. R. Raz, C. K. Lee, L. A. Cannizzaro, P. d'Eustachio, D. E. Levy, Essential role of STAT3 for embryonic stem cell pluripotency. Proc Natl Acad Sci U S A 96, 2846-2851 (1999).
109. G. Reddiconto, C. Toto, I. Palamà, S. De Leo, E. de Luca, S. De Matteis, L. Dini, C. G. Passerini, N. Di Renzo, M. Maffia, A. M. L. Coluccia, Targeting of GSK3β promotes imatinib-mediated apoptosis in quiescent CD34+ chronic myeloid leukemia progenitors, preserving normal stem cells. Blood 119, 2335-2345 (2012).
110. E. P. Reddy, A. Korapati, P. Chaturvedi, S. Rane, IL-3 signaling and the role of Src kinases, JAKs and STATs: a covert liaison unveiled. Oncogene 19, 2532-2547 (2000).
111. R. Ren, Mechanisms of BCR-ABL in the pathogenesis of chronic myelogenous leukaemia. Nat Rev Cancer 5, 172-183 (2005).
112. D. Reynaud, E. Pietras, K. Barry-Holson, A. Mir, M. Binnewies, M. Jeanne, O. Sala-Torra, J. P. Radich, E. Passegue, IL-6 controls leukemic multipotent progenitor cell fate and contributes to chronic myelogenous leukemia development. Cancer Cell 20, 661-673 (2011).
113. A. Rizo, S. J. Horton, S. Olthof, B. Dontje, A. Ausema, R. van Os, V. van den Boom, E. Vellenga, G. de Haan, J. J. Schuringa, BMI1 collaborates with BCR-ABL in leukemic transformation of human CD34+ cells. Blood 116, 4621-4630 (2010).
114. P. Rodriguez-Viciana, P. H. Warne, R. Dhand, B. Vanhaesebroeck, I. Gout, M. J. Fry, M. D. Waterfield, J. Downward, Phosphatidylinositol-3-OH kinase as a direct target of Ras. Nature 370, 527-532 (1994).
115. M. F. Romano, M. Festa, A. Petrella, A. Rosati, M. Pascale, R. Bisogni, V. Poggi, E. C. Kohn, S. Venuta, M. C. Turco, A. Leone, BAG3 protein regulates cell survival in childhood acute lymphoblastic leukemia cells. Cancer Biol Ther 2, 508-510 (2003).
116. J. A. Romashkova, S. S. Makarov, NF-kappaB is a target of AKT in anti-apoptotic PDGF signalling. Nature 401, 86-90 (1999).
117. A. Rosati, M. Ammirante, A. Gentilella, A. Basile, M. Festa, M. Pascale, L. Marzullo, M. A. Belisario, A. Tosco, S. Franceschelli, O. Moltedo, G. Pagliuca, R. Lerose, M. C. Turco, Apoptosis inhibition in cancer cells: a novel molecular pathway that involves BAG3 protein. Int J Biochem Cell Biol 39, 1337-1342 (2007).
118. P. Roychoudhury, R. R. Paul, R. Chowdhury, K. Chaudhuri, HnRNP E2 is downregulated in human oral cancer cells and the overexpression of hnRNP E2 induces apoptosis. Mol Carcinog 46, 198-207 (2007).
119. J. T. Rutka, H. Otsubo, S. Kitano, H. Sakamoto, A. Shirasawa, A. Ochi, O. C. Snead, 3rd, Utility of digital camera-derived intraoperative images in the planning of epilepsy surgery for children. Neurosurgery 45, 1186-1191 (1999).
120. A. Saadatpour, I. Albert, R. Albert, Attractor analysis of asynchronous Boolean models of signal transduction networks. Journal of theoretical biology 266, 641-656 (2010).
121. A. Saadatpour, R. Albert, Discrete dynamic modeling of signal transduction networks. Methods Mol Biol 880, 255-272 (2012).
122. A. Saadatpour, R. Albert, Boolean modeling of biological regulatory networks: A methodology tutorial. Methods 62, 3-12 (2013).
123. M. Saadatpour, A. Afshar, M. H. Afshar, Fuzzy pattern recognition method for assessing soil erosion. Environ Monit Assess 180, 385-397 (2011).
124. D. Sahoo, J. Seita, D. Bhattacharya, M. A. Inlay, I. L. Weissman, S. K. Plevritis, D. L. Dill, MiDReG: a method of mining developmentally regulated genes using Boolean implications. Proc Natl Acad Sci U S A 107, 5732-5737 (2010).
125. M. Sattler, R. Salgia, K. Okuda, N. Uemura, M. A. Durstin, E. Pisick, G. Xu, J. L. Li, K. V. Prasad, J. D. Griffin, The proto-oncogene product p120CBL and the adaptor proteins CRKL and c-CRK link c-ABL, p190BCR/ABL and p210BCR/ABL to the phosphatidylinositol-3' kinase pathway. Oncogene 12, 839-846 (1996).
126. M. Schreiber, A. Kolbus, F. Piu, A. Szabowski, U. Mohle-Steinlein, J. Tian, M. Karin, P. Angel, E. F. Wagner, Control of cell cycle progression by c-Jun is p53 dependent. Genes Dev 13, 607-619 (1999).
127. M. Schuldiner, O. Yanuka, J. Itskovitz-Eldor, D. A. Melton, N. Benvenisty, Effects of eight growth factors on the differentiation of cells derived from human embryonic stem cells. Proc Natl Acad Sci U S A 97, 11307-11312 (2000).
128. J. J. Schuringa, K. Y. Chung, G. Morrone, M. A. S. Moore, Constitutive activation of STAT5A promotes human hematopoietic stem cell self-renewal and erythroid differentiation. J Exp Med 200, 623-635 (2004).
129. J. Seoane, H.-V. Le, J. Massague, Myc suppression of the p21(Cip1) Cdk inhibitor influences the outcome of the p53 response to DNA damage. Nature 419, 729-734 (2002).
130. A. D. Sharrocks, The ETS-domain transcription factor family. Nat Rev Mol Cell Biol 2, 827-837 (2001).
131. A. S. Shet, B. N. Jahagirdar, C. M. Verfaillie, Chronic myelogenous leukemia: mechanisms underlying disease progression. Leukemia 16, 1402-1411 (2002).
132. K. Shuai, J. Halpern, J. ten Hoeve, X. Rao, C. L. Sawyers, Constitutive activation of STAT5 by the BCR-ABL oncogene in chronic myelogenous leukemia. Oncogene 13, 247-254 (1996).
133. K. Shuai, B. Liu, Regulation of JAK-STAT signalling in the immune system. Nat Rev Immunol 3, 900-911 (2003).
134. C. Sillaber, F. Gesbert, D. A. Frank, M. Sattler, J. D. Griffin, STAT5 activation contributes to growth and viability in Bcr/Abl-transformed cells. Blood 95, 2118-2125 (2000).
135. T. Sitnikova, C. Perrone, D. Goff, G. R. Kuperberg, Neurocognitive mechanisms of conceptual processing in healthy adults and patients with schizophrenia. Int J Psychophysiol 75, 86-99 (2010).
136. T. Skorski, P. Kanakaraj, M. Nieborowska-Skorska, M. Z. Ratajczak, S. C. Wen, G. Zon, A. M. Gewirtz, B. Perussia, B. Calabretta, Phosphatidylinositol-3 kinase activity is regulated by BCR/ABL and is required for the growth of Philadelphia chromosome-positive cells. Blood 86, 726-736 (1995).
137. D. D. Spyropoulos, P. N. Pharr, K. R. Lavenburg, P. Jackers, T. S. Papas, M. Ogawa, D. K. Watson, Hemorrhage, impaired hematopoiesis, and lethality in mouse embryos carrying a targeted disruption of the Fli1 transcription factor. Mol Cell Biol 20, 5643-5652 (2000).
138. P. Staller, K. Peukert, A. Kiermaier, J. Seoane, J. Lukas, H. Karsunky, T. Moroy, J. Bartek, J. Massague, F. Hanel, M. Eilers, Repression of p15INK4b expression by Myc through association with Miz-1. Nat Cell Biol 3, 392-399 (2001).
139. L. S. Steelman, S. C. Pohnert, J. G. Shelton, R. A. Franklin, F. E. Bertrand, J. A. McCubrey, JAK/STAT, Raf/MEK/ERK, PI3K/Akt and BCR-ABL in cell cycle progression and leukemogenesis. Leukemia 18, 189-218 (2004).
140. A. Strasser, A. G. Elefanty, A. W. Harris, S. Cory, Progenitor tumours from Emu-bcl-2-myc transgenic mice have lymphomyeloid differentiation potential and reveal developmental differences in cell survival. EMBO J 15, 3823-3834 (1996).
141. A. Sunters, S. Fernandez de Mattos, M. Stahl, J. J. Brosens, G. Zoumpoulidou, C. A. Saunders, P. J. Coffer, R. H. Medema, R. C. Coombes, E. W. F. Lam, FoxO3a transcriptional regulation of Bim controls apoptosis in paclitaxel-treated breast cancer cell lines. J Biol Chem 278, 49795-49805 (2003).
142. J. Tanuma, H. Hiai, H. Shisa, M. Hirano, I. Semba, S. Nagaoka, M. Kitano, Carcinogenesis modifier loci in rat tongue are subject to frequent loss of heterozygosity. Int J Cancer 102, 638-642 (2002).
143. D. Thieffry, R. Thomas, Dynamical behaviour of biological regulatory networks--II. Immunity control in bacteriophage lambda. Bull Math Biol 57, 277-297 (1995).
144. D. Thieffry, R. Thomas, Qualitative analysis of gene networks. Pac Symp Biocomput, 77-88 (1998).
145. E. K. Thomas, J. A. Cancelas, H.-D. Chae, A. D. Cox, P. J. Keller, D. Perrotti, P. Neviani, B. J. Druker, K. D. R. Setchell, Y. Zheng, C. E. Harris, D. A. Williams, Rac guanosine triphosphatases represent integrating molecular therapeutic targets for BCR-ABL-induced myeloproliferative disease. Cancer Cell 12, 467-478 (2007).
146. R. Thomas, S. Mehrotra, E. T. Papoutsakis, V. Hatzimanikatis, A model-based optimization framework for the inference on gene regulatory networks from DNA array data. Bioinformatics 20, 3221-3235 (2004).
147. R. Thomas, C. J. Paredes, S. Mehrotra, V. Hatzimanikatis, E. T. Papoutsakis, A model-based optimization framework for the inference of regulatory interactions using time-course DNA microarray expression data. BMC Bioinformatics 8, 228 (2007).
148. R. Thomas, C. J. Portier, Gene expression networks. Methods Mol Biol 930, 165-178 (2013).
149. L. Tracey, A. Perez-Rosado, M. J. Artiga, F. I. Camacho, A. Rodriguez, N. Martinez, E. Ruiz-Ballesteros, M. Mollejo, B. Martinez, M. Cuadros, J. F. Garcia, M. Lawler, M. A. Piris, Expression of the NF-kappaB targets BCL2 and BIRC5/Survivin characterizes small B-cell and aggressive B-cell lymphomas, respectively. J Pathol 206, 123-134 (2005).
150. M. P. van Iersel, A. C. Villeger, T. Czauderna, S. E. Boyd, F. T. Bergmann, A. Luna, E. Demir, A. Sorokin, U. Dogrusoz, Y. Matsuoka, A. Funahashi, M. I. Aladjem, H. Mi, S. L. Moodie, H. Kitano, N. Le Novere, F. Schreiber, Software support for SBGN maps: SBGN-ML and LibSBGN. Bioinformatics 28, 2016-2021 (2012).
151. S. van Mourik, A. D. van Dijk, M. de Gee, R. G. Immink, K. Kaufmann, G. C. Angenent, R. C. van Ham, J. Molenaar, Continuous-time modeling of cell fate determination in Arabidopsis flowers. BMC Syst Biol 4, 101 (2010).
152. M. T. Veeman, J. D. Axelrod, R. T. Moon, A second canon. Functions and mechanisms of beta-catenin-independent Wnt signaling. Dev Cell 5, 367-377 (2003).
153. M. H. Verheijen, R. M. Wolthuis, L. H. Defize, J. den Hertog, J. L. Bos, Interdependent action of RalGEF and Erk in Ras-induced primitive endoderm differentiation of F9 embryonal carcinoma cells. Oncogene 18, 4435-4439 (1999).
154. A. J. Wagner, M. B. Small, N. Hay, Myc-mediated apoptosis is blocked by ectopic expression of Bcl-2. Mol Cell Biol 13, 2432-2440 (1993).
155. R. S. Wang, A. Saadatpour, R. Albert, Boolean modeling in systems biology: an overview of methodology and applications. Phys Biol 9, 055001 (2012).
156. D. J. Weisdorf, C. Anasetti, J. H. Antin, N. A. Kernan, C. Kollman, D. Snyder, E. Petersdorf, G. Nelson, P. McGlave, Allogeneic bone marrow transplantation for chronic myelogenous leukemia: comparative analysis of unrelated versus matched sibling donor transplantation. Blood 99, 1971-1977 (2002).
157. S. T. Wen, R. A. Van Etten, The PAG gene product, a stress-induced protein with antioxidant properties, is an Abl SH3-binding protein and a physiological inhibitor of c-Abl tyrosine kinase activity. Genes Dev 11, 2456-2467 (1997).
158. G. B. Whitaker, B. J. Limberg, J. S. Rosenbaum, Vascular endothelial growth factor receptor-2 and neuropilin-1 form a receptor complex that is responsible for the differential signaling potency of VEGF(165) and VEGF(121). J Biol Chem 276, 25520-25531 (2001).
159. A. T. J. Wierenga, H. Schepers, M. A. S. Moore, E. Vellenga, J. J. Schuringa, STAT5-induced self-renewal and impaired myelopoiesis of human hematopoietic stem/progenitor cells involves down-modulation of C/EBPalpha. Blood 107, 4326-4333 (2006).
160. K. Willert, J. D. Brown, E. Danenberg, A. W. Duncan, I. L. Weissman, T. Reya, J. R. Yates, R. Nusse, Wnt proteins are lipid-modified and can act as stem cell growth factors. Nature 423, 448-452 (2003).
161. N. K. Wilson, S. D. Foster, X. Wang, K. Knezevic, J. Schutte, P. Kaimakis, P. M. Chilarska, S. Kinston, W. H. Ouwehand, E. Dzierzak, J. E. Pimanda, M. F. T. R. de Bruijn, B. Gottgens, Combinatorial transcriptional control in blood stem/progenitor cells: genome-wide analysis of ten major transcriptional regulators. Cell Stem Cell 7, 532-544 (2010).
162. R. Wisdom, R. S. Johnson, C. Moore, c-Jun regulates cell cycle progression and apoptosis by distinct mechanisms. EMBO J 18, 188-197 (1999).
163. D. Wu, W. Pan, GSK3: a multifaceted kinase in Wnt signaling. Trends Biochem Sci 35, 161-168 (2010).
164. S. Xie, H. Lin, T. Sun, R. B. Arlinghaus, Jak2 is involved in c-Myc induction by Bcr-Abl. Oncogene 21, 7137-7146 (2002).
165. S. Xie, Y. Wang, J. Liu, T. Sun, M. B. Wilson, T. E. Smithgall, R. B. Arlinghaus, Involvement of Jak2 tyrosine phosphorylation in Bcr-Abl transformation. Oncogene 20, 6188-6195 (2001).
166. B. Xu, A. Bhattacharjee, B. Roy, G. M. Feldman, M. K. Cathcart, Role of protein kinase C isoforms in the regulation of interleukin-13-induced 15-lipoxygenase gene expression in human monocytes. J Biol Chem 279, 15954-15960 (2004).
167. E. Yang, J. Zha, J. Jockel, L. H. Boise, C. B. Thompson, S. J. Korsmeyer, Bad, a heterodimeric partner for Bcl-XL and Bcl-2, displaces Bax and promotes cell death. Cell 80, 285-291 (1995).
168. M.-Y. Yang, T.-C. Liu, J.-G. Chang, P.-M. Lin, S.-F. Lin, JunB gene expression is inactivated by methylation in chronic myeloid leukemia. Blood 101, 3205-3211 (2003).
169. G. S. Yochum, C. M. Sherrick, M. Macpartlin, R. H. Goodman, A beta-catenin/TCF-coordinated chromatin loop at MYC integrates 5' and 3' Wnt responsive enhancers. Proc Natl Acad Sci U S A 107, 145-150 (2010).
170. A. Yoshimura, T. Ohkubo, T. Kiguchi, N. A. Jenkins, D. J. Gilbert, N. G. Copeland, T. Hara, A. Miyajima, A novel cytokine-inducible gene CIS encodes an SH2-containing protein that binds to tyrosine-phosphorylated interleukin 3 and erythropoietin receptors. EMBO J 14, 2816-2826 (1995).
171. C. Yost, M. Torres, J. R. Miller, E. Huang, D. Kimelman, R. T. Moon, The axis-inducing activity, stability, and subcellular distribution of beta-catenin is regulated in Xenopus embryos by glycogen synthase kinase 3. Genes Dev 10, 1443-1454 (1996).
172. T. Zarubin, Q. Jing, L. New, J. Han, Identification of eight genes that are potentially involved in tamoxifen sensitivity in breast cancer cells. Cell Res 15, 439-446 (2005).
173. B. Zhang, A. C. Strauss, S. Chu, M. Li, Y. Ho, K.-D. Shiang, D. S. Snyder, C. S. Huettner, L. Shultz, T. Holyoake, R. Bhatia, Effective targeting of quiescent chronic myelogenous leukemia stem cells by histone deacetylase inhibitors in combination with imatinib mesylate. Cancer Cell 17, 427-442 (2010).
174. D. E. Zhang, P. Zhang, N. D. Wang, C. J. Hetherington, G. J. Darlington, D. G. Tenen, Absence of granulocyte colony-stimulating factor signaling and neutrophil development in CCAAT enhancer binding protein alpha-deficient mice. Proc Natl Acad Sci U S A 94, 569-574 (1997).
175. P. Zhang, J. Iwasaki-Arai, H. Iwasaki, M. L. Fenyus, T. Dayaram, B. M. Owens, H. Shigematsu, E. Levantini, C. S. Huettner, J. A. Lekstrom-Himes, K. Akashi, D. G. Tenen, Enhancement of hematopoietic stem cell repopulating capacity and self-renewal in the absence of the transcription factor C/EBP alpha. Immunity 21, 853-863 (2004).
176. C. Zhao, J. Blum, A. Chen, H. Y. Kwon, S. H. Jung, J. M. Cook, A. Lagoo, T. Reya, Loss of beta-catenin impairs the renewal of normal and CML stem cells in vivo. Cancer Cell 12, 528-541 (2007).
177. C. Zhao, A. Chen, C. H. Jamieson, M. Fereshteh, A. Abrahamsson, J. Blum, H. Y. Kwon, J. Kim, J. P. Chute, D. Rizzieri, M. Munchhof, T. VanArsdale, P. A. Beachy, T. Reya, Hedgehog signalling is essential for maintenance of cancer stem cells in myeloid leukaemia. Nature 458, 776-779 (2009).
178. Y. Zhao, B. J. Altman, J. L. Coloff, C. E. Herman, S. R. Jacobs, H. L. Wieman, J. A. Wofford, L. N. Dimascio, O. Ilkayeva, A. Kelekar, T. Reya, J. C. Rathmell, Glycogen synthase kinase 3alpha and 3beta mediate a glucose-sensitive antiapoptotic signaling pathway to stabilize Mcl-1. Mol Cell Biol 27, 4328-4339 (2007).

F. Zindy, P. S. Knoepfler, S. Xie, C. J. Sherr, R. N. Eisenman, M. F. Roussel, N-Myc and the cyclin-dependent kinase inhibitors p18Ink4c and p27Kip1 coordinately regulate cerebellar development. Proc Natl Acad Sci U S A 103, 11579-11583 (2006).
